# Flow-Responsive Noncoding RNAs in the Vascular System: Basic Mechanisms for the Clinician

**DOI:** 10.3390/jcm11020459

**Published:** 2022-01-17

**Authors:** Salvatore De Rosa, Claudio Iaconetti, Ceren Eyileten, Masakazu Yasuda, Michele Albanese, Alberto Polimeni, Jolanda Sabatino, Sabato Sorrentino, Marek Postula, Ciro Indolfi

**Affiliations:** 1 Department of Medical and Surgical Sciences, Magna Graecia University, 88100 Catanzaro, Italy; iaconetticlaudio@unicz.it (C.I.); myasuda9222@yahoo.co.jp (M.Y.); mikelealbanese@gmail.com (M.A.); polimeni@unicz.it (A.P.); jolesbt@hotmail.it (J.S.); sorrentino@unicz.it (S.S.); 2 Department of Experimental and Clinical Pharmacology, Centre for Preclinical Research and Technology CePT, Medical University of Warsaw, 02-097 Warsaw, Poland; cereneyileten@gmail.com (C.E.); mpostula@wum.edu.pl (M.P.); 3 Mediterranea Cardiocentro, 80122 Naples, Italy

**Keywords:** noncoding RNAs, flow, shear stress, vascular remodeling, microRNAs, biomarkers

## Abstract

The vascular system is largely exposed to the effect of changing flow conditions. Vascular cells can sense flow and its changes. Flow sensing is of pivotal importance for vascular remodeling. In fact, it influences the development and progression of atherosclerosis, controls its location and has a major influx on the development of local complications. Despite its importance, the research community has traditionally paid scarce attention to studying the association between different flow conditions and vascular biology. More recently, a growing body of evidence has been accumulating, revealing that ncRNAs play a key role in the modulation of several biological processes linking flow-sensing to vascular pathophysiology. This review summarizes the most relevant evidence on ncRNAs that are directly or indirectly responsive to flow conditions to the benefit of the clinician, with a focus on the underpinning mechanisms and their potential application as disease biomarkers.

## 1. Introduction

The vascular system and the bloodstream interact in several ways. The vessel wall is able to modulate flow conditions but is also influenced by flow [1,2]. In fact, vascular cells sense the changes in the mechanical conditions of their extracellular environment, by means of different mechanisms. Flow sensing elements include transmembrane and cytoskeletal proteins, networks of sugars and ion channels. Hemodynamic forces of blood flow are known to have an important impact on vascular development, function and disease [3,4,5,6]. In fact, increasing evidence now indicates that fluid shear stress (FSS), induced by blood flow, is an essential factor promoting intracellular biochemical signals, and its role in physiological and pathological vascular remodeling has received increasing attention. For example, the observation that flow-mediated hemodynamic forces modulate signaling pathways within several vascular cell types through mechano-transduction has fostered a deeper understanding of the pathogenesis of inflammatory processes underlying atherosclerosis [7,8,9,10]. Among vascular cell populations, endothelial cells (ECs) are perhaps the most sensitive to flow variations. Interestingly, laminar shear stress is a key regulator of arterial endothelium and induces the expression of different molecules to stabilize the endothelial atheroprotective and anti-inflammatory function [11]. Flow conditions are also able to modify cellular lipid composition, thereby influencing biomechanical transduction in response to multiple stressors [12]. Two important examples of transcription factors involved in the atheroprotective effect of fluid shear stress are the flow-dependent Krüppel-like factor 2 (KLF2) [13,14] and nuclear factor erythroid 2-related factor 2 (Nrf2) [15,16]. KLF2 is a negative regulator of inflammation, and its expression is associated with atheroprotective effects via the suppression of pro-inflammatory genes and the upregulation of protective genes. NRF2 is a transcription factor that induces the expression of antioxidant genes in response to oxidative stress. Of note, laminar shear stress can induce expression of both KLF2 and Nrf2 unlike oscillatory shear stress, which suggests that the anti-inflammatory and antioxidative effects of FSS might depend on the regulation of KLF2 and Nrf2 expression [17,18,19]. Unlike physiological shear stress, disturbed flow is characterized by low and/or oscillatory shear stress and is able to actively participate in the development of endothelial dysfunction and atherosclerotic lesions [20,21]. Most studies of mechano-sensing in ECs focus on the role of transmembrane protein complexes on mechano-transduction of hemodynamic forces. ECs have several protein elements such as integrins, intercellular junctional proteins or ion channels that allow the detection of mechanical alterations of single cells, transmission of mechano-transduction from the plasma membrane to the inner cell and between neighbor cells. In vascular endothelial cells, G-protein-coupled receptors (GPCRs) are one of the transmembrane proteins proposed to modulate the response to mechanical stimuli in ECs. In fact, mechanical perturbation of the ECs membrane in response to shear stress leads to ligand-independent conformational transitions in a GPCR [22]. Platelet endothelial cell adhesion molecule (PECAM)-1, VE-cadherin and vascular endothelial growth factor (VEGF) receptors are examples of how shear stress variation is associated with gene expression change in ECs [23]. In fact, these proteins form a mechanosensory complex at cell-cell junctions that is essential for the activation of several shear-sensitive signaling pathways [24].

Similar to ECs, vascular smooth muscle cells (VSMCs) also respond to FSS. On the other hand, L-type calcium channels, the Rho-pathway, and the Notch signaling are among the most relevant signal transduction pathways that mediate the effects of flow alterations on VSMCs [25,26,27,28,29].

## 2. Noncoding RNAs

Noncoding RNA (ncRNA) includes RNAs involved in protein translation, as well as a heterogeneous family of regulatory molecules that are able to modulate gene expression at a post-transcriptional level. NcRNAs are classified into small- and long-noncoding RNAs (lncRNAs) [30,31]. The latter are longer than 200 nucleotides (Table 1). Small RNAs can be classified into PIWI-interacting RNAs (piRNAs), microRNAs (miRNAs) and short interfering RNAs (siRNAs).

**Figure 1:**
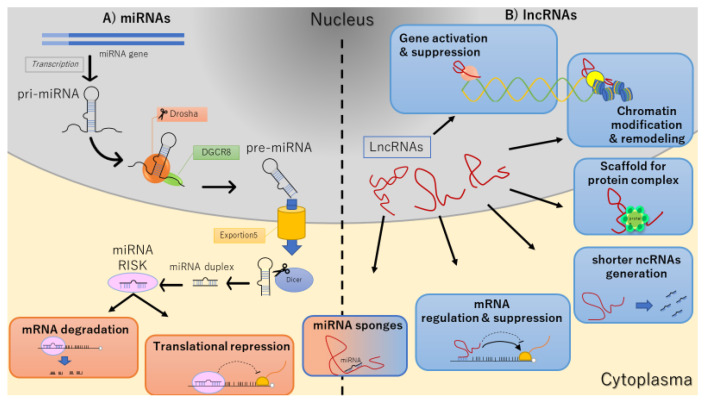
Biogenesis of ncRNAs. The left panel illustrates the main steps of miRNAs genesis and their biological action (**A**). The right panel depicts the origin and the different functions of lncRNAs (**B**).

MiRNAs, are single-strand 18–24 nucleotides, originating from pri-RNA, through clivage by Drosha (ribonuclease III), and DGCR8/Pasha (double-stranded DNA binding protein) [32,33]. MiRNAs bind to target mRNAs, inhibiting their translation or promoting their degradation [34] (Figure 1A). LncRNA are able to exert an array of biological functions, including: (1) regulation of DNA transcription by acting with a transcription factor, (2) epigenetic silencing and repressing the histone modification via chromatin interaction, (3) mRNA translation (4) post-translational regulation through miRNA sponges or circRNA, (5) scaffold of protein complex, (6) shorter ncRNAs (siRNA) generation (Figure 1B).

Noncoding RNAs are largely involved in cardiovascular physiology, atherosclerosis [35], and multiple cardiovascular diseases [36], as chronic and acute coronary syndromes [6,37,38,39,40,41], vascular remodeling [29,42], valvular heart disease [43,44,45,46,47], generation and progression of ectopic calcifications in the cardiovascular system [48,49,50,51,52,53,54], platelet function [55,56,57,58,59], heart failure and stroke [60,61,62,63,64,65,66,67,68,69,70]. Evidence of the impact of flow conditions on circulating ncRNA derives both from observations on pathological changes, such as cardiac valve disease, vascular stenoses or aneurysm, but also from physiological conditions, such as exercise [68].

This review focuses on regulatory noncoding RNAs that are directly or indirectly responsive to flow and their involvement with cardiovascular effects of altered blood flow, with a focus on the potential clinical relevance and the underlying molecular mechanisms and their possible use as clinical biomarkers [71]. As relevant evidence is not equally available for all ncRNAs sub-categories, some specific ncRNAs, including siRNAs, snoRNA or piRNA are not mentioned in this review due to lack of evidence in relation to flow conditions. In addition, the functional category is not known for all regulatory lncRNA to date, as some of them were identified very recently.

## 3. Flow-Mediated ncRNAs

**Figure 2:**
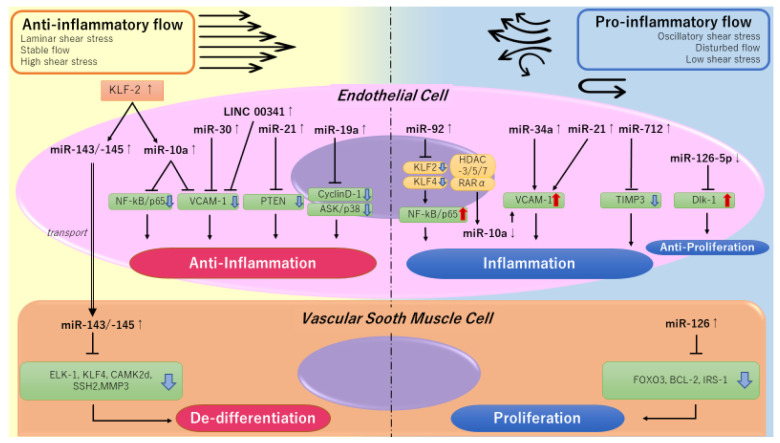
Noncoding RNAs in cells of the arterial wall. The figure depicts the differential anti-inflammatory (**left**) or pro-inflammatory (**right**) effects exerted by selected noncoding RNAs in endothelial cells (**upper figure**) and vascular smooth muscle cells (**lower figure**), in response to different flow conditions.

Endothelial cells (ECs) represent the first interface of the vascular wall with blood. Hence, they are most exposed to all mechanical forces generated by the bloodstream. Several studies described a relationship between flow conditions and the expression of ncRNAs in ECs. Briefly, a disturbed flow (d-flow) and/or unidirectional/oscillatory shear stress (USS/OSS) can induce endothelial dysfunction, thus initiating atherosclerosis by inducing the expression of pro-atherogenic genes, such as VCAM-1 and MMPs. On the contrary, a stable flow (s-flow) or the presence of laminar shear stress (LSS) has an atheroprotective impact, through the activation of a different array of genes, such as Klf2, Klf4, or eNOS [72,73]. Specifically, s-flow/LSS upregulates miR-10a, miR-19a, miR-21, miR-23b, miR-101 and miR143/145, while d-flow/OS upregulates miR-21, miR-34a, miR-92a, miR-663 and miR-712/205 in ECs [74] (Figure 2).

Hence different flow conditions are associated with the expression of specific miRNA patterns in vitro. In line with those findings, several in vivo animal studies showed that artificial d-flow, generated by means of the OSS model, is indeed able to promote atherosclerosis [75]. Experimental modification of shear stress conditions by means of arteriovenous shunts, an established experimental model to promote neoangiogenesis in rats, induces specific modification of miRNA expression patterns leading to the regulation of FOXC1, EPHA2 and SYNJ2BP, thus contributing to flow-induced angiogenesis [76]. Moreover, multiple studies confirmed differential miRNAs expression profiles ex vivo from different regions of the aorta, depending on their exposition to different flow conditions [77,78].

## 4. Flow-Sensitive Anti-Inflammatory miRNAs

### 4.1. miR-10a

The endothelial-enriched miR-10a presents a low expression level in atherosusceptible regions of the aorta, that are exposed to low shear stress (aortic arch, bifurcation of renal aorta), with higher levels in the atheroprotected region (descending aorta, distal renal arteries) in normal adult pigs and rats [77,78]. Expression of miR-10a is induced by LSS/s-flow through inhibition of the anti-inflammatory signal promoted by the nuclear factor kappa-light-chain-enhancer of activated B cells (NF-κB)/p65, a key player in the pathophysiology of atherosclerosis and inflammation [78]. A recent in vitro study indicated that miR-10a expression is regulated by Krüppel-like factor 2 (KLF-2), a known inhibitor of inflammation and NF-κB, through the modulation of retinoid acid receptor-α (RARα) and retinoid X receptor-α (RXRα) [76,77].

Additionally, studies in apolipoprotein E-deficient (ApoE^−/−^) mice showed that miR-10a levels are decreased in aortic endothelium within atherosclerotic lesions [79,80]. Of note, treatment of ApoE^−/−^ mice with RARα/RXRα-specific agonists increased endothelial miR-10a levels and markedly reduced atherosclerosis. These findings are explained by the in vivo induction of endothelial miR-10a, which reduces the GATA6/VCAM-1 pro-inflammatory signals, thereby reducing inflammatory cell recruitment and atherosclerotic lesion formation [17]. Interestingly, it was reported that extracellular vesicles (EVs) secreted from ECs contain miR-10a, and they are transferred to recipient monocytes/macrophages and suppress their activation [81,82]. In this context, miR-10a was shown to suppress NF-κB signaling in monocytes through the repression of Interleukin-1 Receptor-Associated Kinase 4 (IRAK4), MAP3K7, and β-TRC. Consistent with its antiatherogenic role, miR-10a also represses foam cell formation by targeting ligand-dependent nuclear receptor corepressor (Lcor) in macrophages [83,84]. Based on this evidence, shear stress-sensitive miR-10a appears to play a critical role in atherosclerotic lesions.

### 4.2. miR-19a and miR-23b

The regulation of miR-19a levels under LSS conditions was identified in vitro in experiments using human umbilical vein endothelial cells (HUVECs) under LSS [82]. MiR-19a expression was regulated in ECs under LSS, via the suppression of cyclin D1. In turn, miR-19a inhibits ASK1, preventing lipopolysaccharide (LPS)-induced apoptosis [84]. Like miR-19a, miR-23b plays an important role in the flow modulation of endothelial cell proliferation under different flow conditions. In fact, in vitro transcriptional profiling studies of miRNA expression have revealed that miR-23b is upregulated in ECs in response to pulsatile shear flow as compared to the static condition [85]. The mechanisms by which miR-23b regulates ECs growth are a downregulation of cell cycle gene E2F1 and the reduction of Rb protein phosphorylation [85]. The same group also reported that flow-mediated induction of miR-23b is dependent on the expression of the shear stress-induced Krüppel-like factor 2 (KLF2), a key transcription factor that inhibits endothelial proliferation and proinflammatory genes expression [86]. In addition, they provided in vitro and in vivo evidence that pulsatile flow and disturbed oscillatory shear flow differentially regulate miR-23b expression in ECs. Importantly, miR-23b directly targets cyclin H, impair the activity of the CDK-activating kinase complex (CAK), reduces the proliferation of ECs, and inhibits the transcription of cell cycle regulatory proteins [86]. In agreement with this finding, local delivery of miR-23b to the partially ligated vessels inhibited the proliferative ECs phenotype.

### 4.3. miR-143/miR-145

Several studies evaluated the role played by miR-143 and miR-145 in the modulation of VSMCs phenotypic switch through several pathways, including Klf-4, myocd, angiotensin-converting enzyme, and Elk-1 [87]. MiR-145 and -143 (miR-143/145 cluster) are highly expressed in normal arteries and their expression is attenuated in presence of vascular disease. Its modulation has an impact on neointimal proliferation in vivo, as well as in human vascular remodeling in the context of atherosclerosis and aortic aneurysm (AA) [88,89,90,91].

Hergenreider et al. revealed that flow-regulated miR-143 and miR-145 can be transferred from ECs to VSMCs where they are able to induce de-differentiation [92]. Endothelial expression of miR-143 and miR-145 is upregulated in atheroprotected regions of the aorta in vivo as well as in vitro in relation to KLF-2. In addition, the generation of extracellular microvesicles (EMV) containing miR-143/145 is induced by KLF-2 under high shear stress (HSS). Transfer of biologically active miR-143/145 through EMV was demonstrated in co-culture experiments, where the expression level of their target genes, such as ELK1, KLF-4, CAMK2d and SSH2, was reduced in VSMCs after their exposure to ECs that had been previously stimulated to release miR-143/145 enriched EMV [92]. Thus, miR-143/145 expression increase induced under HSS plays an atheroprotective role through EMV-mediated cell-to-cell communication between ECs and VSMCs. These in vitro findings were confirmed by in vivo experiments using ApoE^−/−^ mice, where EMV harvested from KLF2-overexpressing mouse endothelial cells reduced atherosclerotic lesion formation in injected animals compared with controls [92]. Viceversa, miR-143 and miR-145 can be transported from VSMCs to ECs through membrane protrusions known as tunneling nanotubes, a process modulated by the transforming growth factor-β (TGFβ) pathway [93]. In line with this evidence, recent experiments showed that fluid shear stress conditions obtained through femoral artery ligation in rats lead to the upregulation of miR-143-3p, contributing to the reorganization of extracellular matrix through the inhibition of the synthesis of V-α2 collagen [94].

### 4.4. miR-30-5p Family

The miR-30-5p family includes 5 mature miRNA molecules (miR-30a-5p, miR-30b-5p, miR-30c-5p, miR-30d-5p, and miR-30e-5p), which are increased under s-flow/LSS. In vitro experiments using HUVECs and in vivo experiments conducted in mice showed that these changes were associated with a modulation of KLF2 expression [95]. Demolli et al. showed that upregulation of miR-30-5p family members may exert an atheroprotective effect. In fact, the authors reported that overexpression of miR-30a-5p or miR-30b-5p reduced baseline expression and TNF-α-induced expression of E-selectin, ICAM1 and VCAM1. Importantly, miR-30-5p family members also reduced inflammatory cell adhesion to the endothelium, indicating that these miRNAs may have a role in the atheroprotective effects of laminar shear stress. Mechanistically, the miR-30-5p family affects the pro-inflammatory signaling in ECs by targeting the angiopoietin 2 (Ang2), thereby suppressing the expression of adhesion molecules [95].

### 4.5. miR-101

Atheroprotective flow appears to be involved in the regulation of miR-101 expression in ECs. In fact, one study revealed that miR-101 was strongly induced in HUVECs exposed to laminar shear stress [96]. In addition, miR-101 was shown to affect cell proliferation by targeting the 3′UTR of mammalian target of rapamycin (mTOR) in ECs. It is well documented that mTOR is a serine/threonine kinase that regulates cell cycle progression. Repression of miR-101 and consequent upregulation of mTOR contributed to the proliferation of laminar flow-treated ECs [96]. It has been shown that miR-101 plays a key role in angiogenesis. Expression analysis indicated that miR-101 is down-regulated in ECs treated with the pro-angiogenic factor VEGF, and modulated miR-101 expression was described to affect endothelial tubule formation in vitro [97]. Mechanistically, miR-101 directly targets the enhancer of zeste homolog 2 (EZH2) in endothelial cells, leading to inhibition of angiogenesis. In fact, EZH2 knockdown reduced ECs migration, invasion, and tubule formation in vitro and diminished blood vessel formation in glioblastoma tumors in vivo [97]. Importantly, it was found that EZH2 expression is downregulated by atheroprotective shear stress in ECs [98]. In this regard, Xu et al. also reported that miR-101 is involved in laminar flow-induced EZH2 downregulation [99]. In fact, they showed that functional inhibition of miR-101 results in a marked loss of flow-dependent reduction of EZH2 expression.

### 4.6. miR-181

Initially identified as involved hematopoietic-derived tumors [100], members of the miR-181 family were later on associated with cardiovascular diseases, including heart failure [101], stroke [102] and atherosclerosis [103]. More recently, evidence has emerged that shear stress can inhibit the expression of the mechanosensitive miR-181b-5p, that is able to suppress NLRP3 inflammasome-dependent pyroptosis [104]. This latter experimental evidence has recently been mirrored by clinical data revealing that miR-181b acts jointly with the lncRNA ANRIL to mediate the NF-κB signaling, and was therefore proposed as a promising risk biomarker to identify high-risk patients with coronary artery disease (CAD) [105].

## 5. Flow-Sensitive Pro-Inflammatory miRNAs

### 5.1. miR-34a

miR-34a has the ability to induce endothelial senescence by targeting sirtuin 1 (SIRT1) signaling in ECs [106]. In this regard, Tabuchi et al. reported that the levels of miR-34a are increased in endothelial progenitor cells (EPCs) obtained from patients with coronary artery disease (CAD), while SIRT1 levels were reduced [107]. In line with the known involvement of miR-34a in ECs senescence, the qPCR analysis revealed that miR-34a was significantly upregulated in human atherosclerotic plaques [108]. In a study performed on ECs exposed to different shear stress treatments, Fan et al. showed that miR-34a is down-regulated under atheroprotective HSS conditions and upregulated under OSS [109]. The authors also showed that miR-34a binds to a complementary site in the 3′UTR of the SIRT1 mRNA to inhibit its expression and upregulate the activity of the nuclear factor κB (NF-κB), resulting in the enhancement of vascular cell adhesion molecule-1 (VCAM-1) and intercellular adhesion molecule-1 (ICAM-1) protein expression [109]. In agreement with these data, overexpression of miR-34a activates NF-kB signaling and promotes monocyte adhesion to ECs [109]. These data provided evidence for a correlation between the miR-34a expression and flow-dependent regulation of endothelial inflammation.

### 5.2. miR-92a

MiR-92a is a member of the miR-17~92 cluster which is involved in various aspects of vascular disease. Most studies on miR-92a have focused on its role in ECs function. Inhibition of miR-92a significantly enhances endothelial recovery in carotid arteries after balloon injury or arterial stenting [62]. In line with those findings, it has been shown that inhibition of miR-92a in ECs increases endothelial recovery and thus prevents neointimal formation following wire-induced injury in mice [110]. MiR-92a is one of the earliest identified flow-sensitive miRs, targeting factors associated with the atherogenic processes. As mentioned previously, KLF2 can be induced by atheroprotective laminar flow via several mechanisms. Among them, the reduction of miR-92a levels has been depicted. In fact, Wu et al. [111] found that miR-92a was consistently down-regulated in laminar flow-treated ECs and its overexpression in ECs effectively suppressed shear stress induction of KLF2 at both mRNA and protein levels. Importantly, miR-92a represses KLF2 expression by targeting its 3′-untranslated region (3′-UTR). On the contrary, upregulation of miR-92a is associated with decreased mRNA and protein level of endothelial nitric oxide synthase (eNOS) in HUVECs. Consequently, higher levels of miR-92a in mouse carotid artery act to suppress KLF2 activity, and to reduce flow-mediated vasodilation [111]. Subsequent studies showed that miR-92a expression increased in ECs exposed to low shear stress and oxidized low-density lipoprotein (LDL), as well as in atheroprone low shear stress regions of hypercholesterolemic Ldlr−/− mice and in human atherosclerotic lesions [112]. In atherosclerotic mice, functional inhibition of miR-92a is able to prevent endothelial inflammation and atherosclerotic lesions. The effect of miR-92a was mediated by the downregulation of the suppressor of cytokine signaling 5 (SOCS5), KLF2, and KLF4 [112].

### 5.3. miR-712/-205

Using a mouse model of flow-induced atherosclerosis, Son et al. performed miRNA microarray analysis on intimal miRNAs obtained directly from partially ligated left carotid artery or right common carotid artery [74]. The authors then identified miR-712 as an upregulated miRNA closely related to disturbed flow in vivo and in vitro. Further analysis revealed that miR-712 indirectly enhances matrix metalloproteinases (MMPs) activity by directly targeting tissue inhibitors of metalloproteinase 3 (TIMP3), a known inhibitor of MMPs and a disintegrin and metalloproteases (ADAMs). Further, in vivo delivery of Antagomir-712 in a murine model of atherosclerosis increased endothelial TIMP3 levels, decreased endothelial inflammation, and prevented atherosclerotic lesions [74]. It is important to note that miR-712 is mouse-specific. Consequently, whether the role of miR-712 in human vascular disease is played by an equivalent miRNA still remains to be determined. In this regard, Son et al. report that miR-205 is a potential human homolog of murine miR-712. Interestingly, they found that miR-205 expression was upregulated by pro-atherogenic oscillatory shear stress in vitro and in vivo [74]. In line with these data, the expression level of TIMP3 was increased in ECs overexpressing miR-205. Endothelial cells (ECs) represent the first interface of the vascular wall with blood.

## 6. miRNA with Multivalent Effect

### 6.1. miR-21

MiR-21 is upregulated in vitro in HUVECs under LSS conditions [113]. Phosphatase and tensin homology (PTEN), a known target of miR-21, was down-regulated in ECs under LSS or after transfection with the pre-miR-21, resulting in reduced apoptosis, increased endothelial nitric oxide synthase (eNOS) phosphorylation and nitric oxide (NO) production [113]. These data suggest that miR-21 mediates the atheroprotective effect exerted by LSS. On the other hand, miR-21 levels are also increased under OSS conditions, leading to activation of inflammation-related molecules, such as VCAM-1 and MCP-1, via inhibition of peroxisome proliferator-activated receptor-α (PPARα) [114]. Moreover, miR-21 was found to be upregulating in human atherosclerotic plaques, contributing to the progression of atherosclerosis through the induction of VSMCs proliferation by targeting Notch2 and Jag1 [115]. Altogether, these findings suggest that miR-21 is involved in mediating both the atheroprotective effect of favorable shear stress and the pro-atherosclerotic effect exerted by unfavorable flow conditions, depending on the specific biological context. This evidence finds clinical support from recent evidence that miR-21 levels are significantly influenced by the modulation of vascular shear rate during exercise [116].

### 6.2. miR-126

The expression of miR-126 (the standard -3p strand) was found to be 3-fold higher in atherosusceptible aortic arch segments compared with atheroprotected regions of the thoracic aorta in mice, in vivo [117]. Zhou et al. observed that miR-126 was overexpressed in VSMCs after co-culture with ECs that had been previously exposed to OSS [88]. Furthermore, they could demonstrate that suppression of miR-126 expression in vivo using miR-126 knockout mice was able to prevent neointimal proliferation after carotid artery ligation [117]. On the other hand, the overexpression of miR-126 in ECs attenuates TNF-α-induced VCAM-1 expression, reducing leukocyte adhesion to the endothelium [118]. The regulatory proteins Vesicle-Associated Membrane Protein 3 (VAMP3) and Synaptosomal-Associated Protein 23 (SNAP23) are key mediators of vascular dysfunction and associated thrombosis in response to disturbed flow [119]. Both VAPM3 and SNAP23 were recently shown to mediate nonmembrane-bound secretion of miR-126 and its transfer from ECs to VSMCs [120]. MiR-126-5p but not miR-126-3p was down-regulated under d-flow/OSS conditions. Mechanistically, its downregulation is associated with a pro-inflammatory as well as pro-atherosclerotic phenotype [121]. Among the mechanisms underlying these effects, the authors identified the inhibition of delta-like 1 homolog (Dlk1) [122]. Expression levels of miR-126-5p are reduced in human atherosclerotic plaques in areas of disturbed flow. The lower levels of miR-126-5p partially release the inhibitor effect exerted on caspase-3, increasing the risk of adverse plaque remodeling and contributing to the impairment of autophagy [123,124].

## 7. Flow Conditions and Arterial Remodeling

Changes in shear stress can modulate the expression of miRNAs which are able to modulate arterial remodeling, including control of inflammation in ECs and macrophages, as well as the regulation of the VSMCs’ phenotype after vascular injury [125]. ECs can sense flow conditions, that regulate the expression of several atheroprotective genes, such as KLF2. On the contrary, reduced shear stress shifts ECs towards a pro-atherogenic phenotype [126]. Of note, miRNAs modulate ECs’ response to shear stress. Among the others, miR-19a was identified as a flow-responsive miRNAs in ECs [82,91]. In an in vivo study, the potential contributions of regulatory miRNAs within regions of susceptibility to atherosclerosis were investigated by artery site-specific miRNAs profiling in adult swine [78]. This approach revealed that expression of the endothelial miR-10a was less pronounced in the atherosusceptible regions of the inner aortic arch and aorto-renal branches, compared to other regions. In cultured human aortic ECs, it was observed that miR-10a inhibits inflammatory signals acting on multiple components of the IkB/nuclear factor-kappa B (NF-kB) pathway [81]. Although no direct evidence is available on the specific effects exerted by shear stress on vascular remodeling after angioplasty, it is known that any perturbation of tissue stretch can influence its biological homeostasis with consequent effects on vascular remodeling [127].

In a recent study on human umbilical vein endothelial cells (HUVECs) exposed to unidirectional shear stress (USS), 13 upregulated miRNAs were identified, of which miR-21 had the greatest fold-change [113]. MiR-21 inhibits ECs migration via repression of RhoB [128]. As described in the previous paragraph, it should be noted that miR-21 exerts multiple effects on the vessel wall upon variations in flow conditions. As reported above, members of the miR-17/82a cluster are regulated by flow conditions and might exert a pro-inflammatory effect on the vessel wall. According to a recent study, members of the miR-17, miR-19 cluster were down-regulated upon pulsatile flow [85]. In contrast, laminar shear stress-induced miR-19a expression in ECs. The latter is a key modulator of cyclin D1 and endothelial proliferation in response to changes in flow [82]. Furthermore, miR-92a expression was inhibited by laminar flow and upregulated by oscillatory flow, while miR-27a and miR-27b were highly expressed in ECs, where they play a key role. For instance, miR-27b was down-regulated upon silencing of Dicer or Drosha, resulting in a significant reduction of ECs sprouting in vitro [129]. In addition, miR-27a/b promotes angiogenesis by binding to the 3′-untranslated regions (3′-UTRs) of the angiogenesis inhibitor SEMA6A, which controls the repulsion of neighboring ECs [129]. More recently, the shear-stress modulated miR-27 was shown to promote the interaction of ECs with pericytes, improving the barrier function of the endothelium in a mouse model [130].

In a mouse model of hyperlipidemia, miR-155 expression was increased in vivo by acutely disturbed blood flow [131]. In turn, miR-155 exerted a pro-inflammatory role on lesion macrophages, leading to the progression of atherosclerotic lesions [131]. On the other hand, in apparent contrast with the results described above, a different study showed that miR-155 exerts an anti-inflammatory effect, reducing atherosclerosis progression in hyperlipidemic mice without disturbed blood flow [132].

The upregulation of miR-633 under OSS conditions was demonstrated in a microarray analysis on HUVECs exposed either to oscillatory shear stress (OSS) or laminar shear stress (LSS) [133]. In the same study, antagonization of miR-663 was able to inhibit monocyte adhesion, whereas its over-expression induced by LSS increased monocyte adhesion in vitro [133]. MiR-663 targets several transcription factors, such as KLF4, CEBPB and ATF3, which are key regulators of inflammation and atherosclerosis. Inhibition of miR-663 partially restores KLF4 expression in ECs under OSS, suggesting that OSS-induced miR-663 is a “fine-tuner” of KLF4 expression in ECs [134]. The overexpression of miR-663 in VSMCs increased the expression levels of known proliferation and migration marker genes [135].

## 8. miRNAs and Arterial Aneurysms

Arterial aneurysms can be the result of an imbalance of vascular remodeling. In line with this hypothesis, some of the miRNAs that are known to be involved in vascular remodeling such as miR-21, miR-24, miR-126, miR-155, miR-205, miR-712, miR-26a, miR-143/145, miR-29, and miR-195 have been recently associated with the development of aortic aneurysm (AA). Among these, some miRNAs are sensitive to flow conditions, as described in the previous paragraphs.

For example, miR-126 is involved in cell migration and blood vessel formation and is sensitive to flow conditions [136,137]. More recent studies showed that miR-126 is a key element to maintaining vascular integrity. In fact, knockout of miR-126 in mice and zebrafish decreases vascular integrity and impairs proliferation, migration, and angiogenic activities of ECs [138,139]. Furthermore, downregulation of miR-126 in plasma and upregulation in abdominal aortic aneurysmal tissues have been observed and indicate the potential role of miR-126 in AA formation, suggesting that the regulatory impact of miR-126 on vascular inflammation and remodeling is closely related to the development of aortic aneurysms [140,141,142].

A recent ex vivo study revealed significant upregulation of miR-155 in human abdominal aortic aneurysms (AAA) as compared to non-dilated aortic segments from the same patient [143]. In the same samples, the overexpression of miR-155 was associated with a significant reduction in the expression levels of cytotoxic T-lymphocyte-associated protein (CTLA4) and SMAD2, known targets of miR-155 [143]. The changes observed in miR-155 levels could be at least in part related to the altered flow conditions around the aneurysm. In this regard, in vitro studies reported that miR-155 mediates profound remodeling of the cytoskeleton in the aortic wall of mice [144].

It is well known that miR-21 is involved in several mechanisms underlying vascular remodeling. In fact, it is able to promote differentiation of VSMCs in response to transforming growth factor-β (TGF-β) and bone morphogenetic protein (BMP). These effects are dependent upon the decrease in PDCD4 expression and finally result in the inhibition of apoptosis through the downregulating of PTEN and the upregulating BCL2 [145]. More recently, Maegdefessel demonstrated that miR-21 overexpression inhibits apoptosis and downregulates PTEN in the aortic wall in vivo and prevents AA expansion [146]. Conversely, antagonization of miR-21 by systemic injection of a locked nucleic acid-(LNA-) modified antagomir targeting miR-21 was associated with a marked increase in the size of AA, suggesting that miR-21 is a critical element in the development of AA [146].

The miR-143/145 cluster is one of the most studied in VSMC, as it plays a key regulatory role in this cell population. In particular, it is a key regulator of the phenotypic switch between a contractile and a synthetic phenotype [87,88,89,90,91]. Recent studies revealed that the transition of VSMCs of the tunica media from a contractile to a synthetic phenotype is associated to an increased risk for thoracic aortic dissection (TAD) [147]. In a similar background, Liao and colleagues observed that miR-143/145 expression is lower in the context of TAD lesions, which further supports the hypothesis that VSMC under differentiation contributes to the development of TAD [148]. In line with this hypothesis, an inverse correlation was found between the expression levels of miR-143/145 and the degree of dedifferentiation of VSMCs [148]. A very recent study reports that miR-145, together with other aneurysm-enriched miRNAs participate in the development of AA [140]. Most interestingly, specific patterns of miRNAs modulation were observed for toracic AA (miR-1, -29a,133a, and -221) and abdominal AA (miR-145, -146a, -331) [139].

## 9. Long Noncoding RNAs Responsive to Flow Variations

An increasing number of studies have shown that lncRNA expression can be regulated by a flow-dependent mechanism. More recently, the newly annotated long noncoding RNA MANTIS (lncRNA n342419) was shown to modulate the impact of shear stress on angiogenic sprouting or alignment of endothelial cells [149]. qRT-PCR measurements have revealed that MANTIS is upregulated in ECs in response to laminar flow as compared to static conditions. Interestingly, MANTIS was able to interact with BRG1, thus allowing its ATPase function, finally resulting in the transcription of key genes involved in endothelial function and angiogenesis, such as SOX18, SMAD6, and COUP-TFII [149,150]. MANTIS then functions as a mediator for chromatin remodeling complexes and enhances endothelial angiogenic function.

The lncRNA spliced-transcript endothelial-enriched lncRNA (STEEL) is enriched in ECs and its levels are sensitive to flow conditions. It has been recently shown to interact with multiple miRNAs [151]. To identify functionally significant lncRNAs in vascular endothelium, Man et al. performed a microarray analysis of different cell types, and they observed a high expression of STEEL in ECs [152]. They also observed that the expression of STEEL increased in ECs exposed to atheroprotective flow. Importantly, gain-of-function and loss-of-function experiments showed that STEEL significantly promotes ECs migration, network formation in vitro, and blood vessel formation in vivo. Mechanistically, STEEL mediates its biological functions at least in part by promoting the transcription of endothelial nitric oxide synthase (eNOS) and KLF2. Additional recent evidence showed how favorable shear stress upregulates the lncRNA AF131217.1 in human umbilical vein endothelial cells (HUVECs). This lncRNA acts as an endogenous competing RNA for miR-128-3p, ultimately resulting in the regulation of KLF4 and KLF2 [153].

In a recent report, Miao et al. performed RNA-seq analysis to investigate lncRNA expression in HUVECs exposed to physiological or pathological flow conditions [154]. Among the lncRNAs differentially expressed, they identified and characterized “lncRNA that enhances eNOS expression” (LEENE) as a potential regulator of endothelial function and strongly correlated to eNOS expression in response to pathological flow. The authors found that inhibition of LEENE not only causes the transcription of pro-inflammatory molecules but also suppresses eNOS mRNA and protein expression in HUVECs. Importantly, LEENE promotes eNOS nascent mRNA transcription by the recruitment of RNA Pol II to the eNOS promoter [154].

Another interesting lncRNA is the endothelial cells enriched LINC00341. Among the most abundant in endothelial cells, it is an important modulator of endothelial function in response to pulsatile shear flow patterns [155]. LINC00341 is able to inhibit the adhesion of monocytes via downregulation of vascular cell adhesion molecule-1 (VCAM-1). In addition, it can also exert an antiinflammatory effect, guiding the enhancer of zeste homolog 2 (EZH2) to the VCAM-1 promoter [155]. Finally, LINC00341 is able to modulate both the Rho- and the PI3K/AKT-signaling pathways [155].

The lncRNA antisense noncoding RNA in the INK4 locus (ANRIL) is an important regulator of cell proliferation and senescence and was recently associated with atherosclerosis and cardiovascular diseases [156]. In fact, multiple reports have demonstrated that ANRIL is able to regulate key modulators of VSMCs’ and ECs’ migration and differentiation, such as CDKN2a, CDKM2B, DAB2IP, LRP1, LRPR and CNTN3 [157,158,159,160].

Another flow-sensitive lncRNA is long intergenic noncoding RNA antisense to S1PR1 (LISPR1). Its expression levels were found to be significantly higher in HUVEC after exposure to laminar shear stress [161]. Moreover, LISPR1 was shown to be a positive regulator of Sphingosine-1-Phosphate (S1P) signaling and endothelial function. In fact, knockdown of LISPR1 decreased S1P receptor1 (S1PR1) expression and selectively disrupt the S1P downstream signaling in ECs, impairing migration and spheroidal ECs outgrowth [161].

Angiotensin II and Urotensin II, able to modulate vascular flow through their vasoconstrictive capacity, are associated with the risk to develop cardiovascular disease [23,162]. Very recently, the lncRNA LncAng362 was shown to be a regulator of cell response to angiotensin II. Interestingly, LncAng362 mainly exerts its function modulating the expression levels of two key miRNAs involved in the regulation of VSMCs proliferation, such as miR-221 and miR-222, providing a further example of complex interaction networks between different classes of noncoding RNAs [163].

The lncRNA HIF 1 alpha-antisense RNA 1 (HIF1a-AS1), is another recent discovery. It is an emerging regulator of both vascular remodeling and VSMCs’ apoptosis and proliferation, in response to flow conditions. Accordingly, it was associated both with neointimal proliferation and aneurysm formation [164,165].

Another vascular cell-enriched lncRNA implicated in ECs function is SENCR, which plays a pivotal role in multiple cellular processes including cell migration, cell differentiation, and angiogenesis. In this regard, a recent in vitro and in vivo study also identified SENCR as a flow-responsive lncRNA [166]. Of note, Lyu et al. showed that SENCR was upregulated in endothelial cells stimulated by laminar shear stress. These in vitro findings are consistent with in vivo analyses showing that the laminar shear stress region of the adult aorta of humanized SENCR-expressing mice have increased SENCR levels when compared with those of the disturbed shear stress regions. In addition, Lyu et al. produced a lentivirus that downregulated SENCR expression and induced membrane permeability of laminar shear stress-stimulated ECs through a decrease in CDH5 protein at cell–cell junctions, suggesting a potential role of SENCR in the regulation of membrane integrity. The author also performed a biotinylated RNA pull-down assay in static ECs and showed that SENCR binds cytoskeletal-associated protein 4 (CKAP4). They then speculated that the function of SENCR in membrane permeability and integrity of ECs could be mediated through the binding to noncanonical RNA-binding protein CKAP4. In fact, CKAP4 binds CDH5 resulting in CDH5 internalization at the adherens junction. SENCR is then required to regulate the ECs membrane homeostasis is laminar shear stress by forming a ribonucleoprotein complex with CDH5 [166].

Recent experiments in mice models showed how the protective role of laminar flow on the vascular endothelium is at least in part exerted through the facilitation of nuclear localization of nesprins, which increase the possibility for beta-catenin to access the nucleus where it can increase the transcription of the lncRNA MALAT1 with a positive impact on tight junctions and cellular barrier functions [167]. In addition to tight junctions, adherens junctions also play an important role in maintaining the endothelial barrier junction. They are stabilized by shear stress through the increased expression of the lncRNA LASSIE [168].

## 10. Conclusions

A growing body of evidence has been accumulating on the involvement of ncRNAs in the modulation of several biological processes linking flow-sensing to vascular pathophysiology, suggesting some potential new target sites for novel therapeutic strategies in the future. A number of noncoding RNAs that are measurable in the blood are directly or indirectly involved in the biological mechanisms underlying flow-sensing and the modulation of its impact on different cells and tissues. Hence, they are a potential source of new “smart” biomarkers, given their peculiarity to reflect the pathophysiological mechanisms underlying the disease or biological process to be monitored or diagnosed. Clinicians should be aware of this uprising field, as the potential clinical use of these ncRNAs as biomarkers might become a reality in the very next future.

## Figures and Tables

**Table 1:** Classification of non-coding RNAs.

Housekeeping ncRNA	Symbol	Function
	rRNA (ribosomial)	mRNA translation
	tRNA (transfer)	mRNA translation
	snoRNA (small nucleolar RNA)	RNA modification, rRNA procesing
**Regulatory ncRNA**	**Symbol**	**Function**
Short ncRNA (<200 nt)	piRNA (PIWI-interacting RNA)	DNA methylation, transposition repression
	miRNA (microRNA)	Transcriptional regulation
	siRNA (short interfering RNA)	RNA interference
lncRNA (>200 nt)	lincRNA (long intergenic RNA)	Epigenetic regulation of transcription
	eRNA (enhancer-like ncRNA)	Transcriptional gene activation
	T-UCR (transcribed ultraconserved regions)	Regulation of miRNA and mRNA levels
	NAT (natural antisense transcripts)	mRNA stability
	PALR (promoter-associated long RNA)	Chromatin changes
